# The Repetition of the Dose

**Published:** 1890-05

**Authors:** Ad. Lippe


### THE REPETITION OF THE DOSE.

DR. AD. LIPPE.

It has been often asked by beginners of the practice of Homœopathy, as well as by students, how often a dose of medicine should be repeated. *A priori*, no rules for the repetition of the dose can be laid down. In very acute diseases, one single dose may suffice, or it may be necessary to repeat the dose at very short intervals; in chronic diseases, one dose may act for days, weeks, and months, or it may become necessary to repeat the dose daily or oftener for a day, a week, or even for months. In all this the practitioner must be guided by his individual judgment. Individual judgment must not be mistaken for, or confounded with, individual opinion, individual whim, or individual caprice; these mistaken notions of inalienable rights to indulge in a licentious freedom of medical opinion and action are adverse to the scientific and sure guidance to which individual judgment submits. Individual judgment implies in this, as in every case in which a practical application of fundamental laws and rules is to be made, that the practical application left to the individual judgment of the practitioner of a science must positively be in harmony with the laws governing that science, and with the rules already established governing their practical application. In chemistry, as well as in all scientific pursuits, fundamental rules and laws exist which must be followed, if the investigator expects to reach or obtain satisfactory results.

The individual judgment implies, therefore, that the practitioner has to judge in every given case for himself how previously established laws and rules, which he is supposed to have accepted when he attempts to practice, shall and must be applied. It will be clear to his mind that the very first rule ever laid down by HAHNEMANN, and accepted by his followers, respecting the repetition of the dose, is "*The dose must not be repeated till the action of the last dose administered has been fully exhausted.*" Accepting this as a sure guide, other questions present themselves to the thinking practitioner.

1st. What are the infallible indications showing the favorable action of a dose administered?

2d. What are the infallible indications that its action has been exhausted?

After the administration of a properly potentized homœopathic remedy, given singly and in a single dose, we see its effect in an acute disease very soon, often in a few minutes, and the more acute and the more severe the attack, the sooner may the development of the action of that dose be expected. The close observer will perceive, very soon after the administration of the dose, some auspicious symptoms showing him the action of the dose administered. Great distress and pains may suddenly, and for a short time, be aggravated, or may cease and sleep set in, or the stomach be relieved of its contents when it had been overloaded and suffering was caused by it, or mental anguish give place to quietude, the pulse may change for the better, the thermometer may show an improvement. If the action of the dose administered has once begun, and if even the improvement is slow but steady, then we know that the dose administered continues to develop its curative powers, or we may infer that the *vis medicatrix naturæ* once set to develop its health-restoring office, is still at work, and wants no other aid by medicine. In chronic diseases the action of the dose administered cannot develop such sudden effects; this would be contrary to the nature of a long-existing and deep-seated disease. If such a sudden exhibition of the drug-action follows its administration; if the improvement of the case is very rapid, then either the remedy acted as a palliative only, or was not rightly chosen; or, if very similar and carefully chosen, such sudden improvement *generally* forebodes no good, a repetition rarely ever produces a perceptible improvement, and other ever so well-chosen remedies will cause rapid but short-lasting improvement. It is especially in chronic diseases that aggravations frequently follow the administration of a truly homœopathic remedy, and if new symptoms appear, of which the sick complained previously, then we may infer with almost positive certainty that the remedy is developing its curative powers. A very perceptible improvement, such as is acknowledged by the sick himself, very frequently does not take place in acute diseases before the third day; this is to be accounted for, not by any pathological deductions, but by the fact that the sick-making powers of a single dose of a well-potentized drug, when taken by a healthy person, very frequently do not begin to show their effects until the third day after it has been taken; the very attentive observer will in such cases have perceived very soon after its administration to the healthy the same auspicious symptoms he has learned to observe on the sick. A repetition of the dose before the one previously administered has developed its effects, or before its effects are exhausted, causes an interruption of the internal, to our perceptions and understandings, hidden, process in the interior of the organism, having for its object the restoring of the sick to health, therefore must be avoided; and, furthermore, such an untimely interference is invariably followed by results retarding a recovery, and may even at times so derange the action of the organism, striving to combat the existing disturbances, that the recovery may not only be retarded, but be made very doubtful.

We know that the curative powers of a dose administered have been exhausted when the improvement comes to a perfect standstill, especially in acute diseases; a repetition of the same remedy may become necessary if the existing symptoms still indicate it. It was HAHNEMANN who advised us, in his *Chronic Diseases*, then to administer a *different potency*, but if new or other symptoms present themselves, then *another remedy* has to be chosen. In chronic diseases especially will it happen that the symptoms for which the remedy has been administered have been entirely removed, but that in the course of time, often after some weeks, the same previously observed symptoms reappear in a modified form ; in this case the action of the formerly administered dose continues, and a repetition would materially interfere with the cure. This can be accounted for by the fact, that persons who have suffered from a succession of symptoms from a single dose administered, found these symptoms disappear for a time, but that after days and sometimes after weeks the same symptoms reappeared in a modified form, without a repetition of the dose of the drug first taken. If a repetition of the dose becomes necessary because the effects of the last dose administered have been fully exhausted, it must again be left to the individual judgment of the physician in what manner this repetition should be made. If a dose administered has acted for a long time, in acute diseases for days, in chronic diseases for weeks or months, we may reasonably judge that it would be best to again administer one more single dose ; but if the action of the dose has lasted only a comparatively short time, has been rapidly exhausted, especially in acute diseases, and a repetition appears still advisable, then it would almost always be better to dissolve a single dose of the remedy now to be repeated in some few ounces of water, and continue its administration in broken doses till it becomes evident that the action of the dose in this manner administered has fully set in, and the symptoms for which it was given are yielding to it, becoming lessened in every respect; in chronic diseases, the individual judgment of the physician may lead him to administer the remedy in daily doses or in many doses a day for a length of time, till it becomes evident that the symptoms are materially relieved, and then the action of the repeated doses will scarcely ever be exhausted in a short time, but will probably last for weeks and months.

The greatest care should be taken never to repeat the dose, or administer another remedy till the effects of the dose last taken have been exhausted. This dose may be, and often is, a single dose, or it may be a dose dissolved in water and given at short intervals, or it may be a repetition of doses at short intervals, till some effect of this dose is apparent.

[The foregoing article from the pen of our lamented master and friend, the late Dr. Adolph Lippe, first appeared in 1878 in the pages of a journal called the *Organon*, published by the distinguished Dr. Thomas Skinner, now of London. It may be found at page 286 of volume I.

We consider the present a most fitting time for the republishing of this article, as the question which it treats was discussed in the International Congress, whose proceedings are given in our April number. It seems a comprehensive answer to the ideas advanced in the sessions of the Congress. To many of our readers it is new, and we are sure all will welcome it as being most instructive, and a reminder of one of the foremost men of the homœopathic school.—W. M. J.]

